# The Impact of an 8-Week Running Technique Program on Linear and Change-of-Direction Speed in Youth Football—A Pilot Study

**DOI:** 10.3390/sports13090305

**Published:** 2025-09-04

**Authors:** Diogo Camacho, Diogo Monteiro, Rui Matos, Nuno Amaro, Raúl Antunes, Miguel Jacinto

**Affiliations:** 1 ESECS—Polytechnic University of Leiria, 2411-901 Leiria, Portugal; 1220765@my.ipleiria.pt (D.C.); diogo.monteiro@ipleiria.pt (D.M.); rui.matos@ipleiria.pt (R.M.); nuno.amaro@ipleiria.pt (N.A.); raul.antunes@ipleiria.pt (R.A.); 2 Research Centre in Sports Sciences, Health Sciences and Human Development (CIDESD), 6201-001 Covilhã, Portugal

**Keywords:** athletic development, change of direction, football (youth), injury prevention, linear speed, running technique, sports safety

## Abstract

In football, linear speed and change-of-direction speed are fundamental skills for performance in the sport. The present study aims to evaluate the effect of an 8-week running technique program on the variables described in young footballers. Thirty-one athletes participated, 16 in the intervention group (Under-15) and 15 in the control group (Under-17). The intervention group had a mean age of 14.37 ± 0.50 years and the control group had a mean age of 15.80 ± 0.76 years. Both groups underwent two assessments, pre- and post-intervention, performing the 20-m test and the 5-0-5 Agility Test, assessing linear speed and change-of-direction speed, respectively, with timing conducted manually by trained evaluators. The results showed improvements in the intervention group in both tests, with statistically significant differences in change-of-direction speed (right foot: *p* = 0.010; r = 0.669; left foot: *p* = 0.05; r = 0.503), while the control group did not show any significant differences in either test, even showing a regression in results. The present study indicates that running technique training, even with a weekly frequency of only one session per week, may contribute to improvements in linear speed (even non-significant) but, especially, in the change of direction of young footballers. These results are important because training can promote more efficient running movement patterns, promoting benefits in terms of sports performance and the prevention of non-contact injuries.

## 1. Introduction

Football is an intermittent, high-intensity sport with complex physical and cognitive demands. During the 90 min of a game, a professional player runs an average of 10 to 12 km, alternating between submaximal efforts and short bursts of explosive action [1]. These actions include sprints, accelerations, changes of direction, jumps, and physical contact, requiring not only physical qualities, such as strength, power, and endurance, but high motor and technical skills [2,3].

Among the most decisive physical components for performance in modern football are linear speed and the ability to change direction. Studies have shown that a player performs a sprint, on average, every 90 s, with 96% of these sprints covering less than 30 m [1]. In addition, according to Faude et al. [4], 51% of the goals scored in the German first division resulted from linear sprinting or changes of direction immediately before the finish or assist. These data show that speed and agility not only directly influence offensive success but are essential for defensive tasks, quick transitions, and tactical imbalances.

Sprinting can be divided into two biomechanically distinct phases: the acceleration phase and the maximum speed phase [5]. During acceleration, the trunk is more inclined, the ground contact time is longer, and the application of force is predominantly horizontal. As the athlete reaches maximum speed, the posture becomes more upright, the ground contact is reduced, and the efficiency of the stretch-shortening cycle becomes crucial for maintaining speed. The efficient execution of this movement depends on factors such as intermuscular coordination, tendon stiffness, neuromuscular activation pattern, and running technique [6,7].

The technical quality of running thus plays a central role in optimizing performance. Poor technique can limit the expression of strength and power, reduce the running economy, and, above all, increase the risk of muscle injuries, especially in the hamstrings, which are often overloaded during sprinting [8]. Improving running techniques through specific exercises that correct posture, limb positioning, and ground contact pattern can simultaneously enhance performance and act as a preventive tool.

This aspect is particularly relevant in youth football, where athletes are in phases of accelerated growth, with imbalances between strength, coordination, and motor control. Epidemiological studies have shown that non-contact injuries account for more than 58% of injuries in elite young football players, predominantly affecting the lower limbs [9]. These patterns suggest that changes in neuromuscular control and running mechanics may be risk factors, especially during peak growth phases, where maturation may not keep pace with training or competition loads [10].

The recent literature points to the potential of running technique programs to improve physical performance. Lupo et al. [11] demonstrated that young football players who underwent 12 weeks of technical training significantly improved their times in sprint and agility tests with the ball, compared to a group that underwent conventional technical–tactical training. Similarly, Martín-Moya et al. [12] showed that combining running technique with strength and plyometrics resulted in significant improvements in change of direction and lower limb power. In addition to performance benefits, Silva et al. [8] showed that a 6-week running technique program promoted protective biomechanical changes in sprint patterns, with a possible impact on reducing the risk of hamstring injury.

Within the young population of football players, linear speed and change-of-direction speed are two performance aspects that are very important. However, youth athletes are still developing both physically and psychologically and could account for some positiveness in long-term performance influences. Biological maturation can certainly influence neuromuscular adaptations and reflexive responses where they could be hurrying an accommodative response after peak growth velocity, for example, during the adolescent growth spurt, thus stimulating bigger or smaller training responses [13]. For example, early maturing players could demonstrate some performance benefits because of accelerated strength gains. Late maturing players could display these performance improvements but on a delayed trajectory, potentially demonstrating the best adaptations [10]. Only a few studies have investigated such growth-related maturational variables, generating challenges in potential generalisability relating to short-term intervention-related outcomes. Therefore, to quantitatively assess training effects in young athletes, it is important to consider chronological age and biological maturation status, so as researchers we can distinguish actual training adaptations and learning from natural growth patterns.

Despite growing international evidence, few studies have focused on pre-pubertal athletes. The maturational state must be considered when analysing performance adaptations in youth populations. There are still few studies addressing running technique as a development tool in young athletes, especially during the pre-pubertal ages or at the beginning of athletic maturation. Gil et al. [14] addressed the impact of running technique on players between the ages of 18 and 23, but research in younger age groups remains limited. The results of this study may provide new insights into how structured running technique training can be optimized for pre-pubertal and early maturing athletes, a population often overlooked in the existing literature. If demonstrated to yield measurable improvements in sprint performance and agility through targeted drills, our results could provide evidence-based guidelines for coaches working with young athletes during critical developmental windows.

Thus, the present study aims to evaluate the effects of an 8-week running technique program on linear speed and change-of-direction speed in young football players. We hypothesize that the intervention group will improve sprint and agility performance relative to the control group and/or pre-test.

## 2. Materials and Methods

### 2.1. Participants

This study initially involved 31 male football players (Tier 2: Trained/Developmental [15]), divided into two youth teams: Under-15 (*n* = 16) and Under-17 (*n* = 15). The distribution of the groups was defined in conjunction with the club, and it was decided, for convenience and logistics, that the intervention group would consist of athletes from the Under-15 team and the control group would consist of athletes from the Under-17 team.

The anthropometric characteristics of the participants were recorded at the beginning of this study. The intervention group (Under-15) had a mean age of 14.37 ± 0.50 years, a mean body weight of 59.44 ± 10.55 kg, and a mean height of 171 ± 10.5 cm. The control group (Under-17) had an average age of 15.80 ± 0.76 years, an average body weight of 66.3 ± 6.14 kg, and an average height of 174 ± 5.3 cm.

To be included in this study, athletes had to meet the following eligibility criteria: (i) participate in both pre- and post-intervention assessments; (ii) attend at least 6 of the 8 sessions of the running technique program. At the same time, to be included in this study, athletes had to meet the following eligibility criteria. Athletes with attendance below 75% were excluded, which is consistent with protocols in pilot field interventions. Participant attendance at the sessions was monitored using a standardized attendance sheet, where each athlete confirmed their presence at the end of each session, thus ensuring control of exposure to the intervention. Athletes who did not meet the inclusion criteria, namely absence from assessments or failure to meet the minimum attendance requirement for training sessions, were excluded from the final analysis. As a result, the final sample consisted of 30 athletes, 15 in the intervention group (Under-15) and 15 in the control group (Under-17).

Despite the age difference between the groups, it was decided to maintain this group configuration, due to logistical constraints imposed by the club, which resulted in age group differences, recognizing this characteristic as a potential limitation of sample homogeneity in terms of biological and maturational development.

A power analysis (calculated using G*Power, version 3.1.9.7) showed that a sample of at least 15 (for each group) was required to detect a medium effect size (ES) of 0.85 (*α* = 0.05, 1 − *β* = 0.7), for the Wilcoxon–Mann–Whitney test (two groups). An effect size of 0.5 was chosen due to the lack of studies of this nature.

### 2.2. Instruments

Two tests were used: a 20-m sprint and the “5-0-5 Agility Test”, where the first seeks to assess linear speed and the second assesses speed of change of direction.

Before the assessment, all participants performed a general warm-up led by their respective coaches. The warm-up included general mobility exercises and dynamic stretching, which are common in each team’s training sessions. After the warm-up, they were gathered and introduced to the test procedures.

#### 2.2.1. Linear Speed—20 m

Linear speed was assessed using the 20-m sprint test, one of the most widely used and validated tools in youth football to measure acceleration and maximum speed over short distances [16].

The test was set up on an artificial turf field, using four markers, a measuring tape, and a precision digital stopwatch. The course was set at a total distance of 20 m in a straight line.

Each athlete performed two attempts, with a 1- to 2-min passive rest interval between repetitions, to ensure recovery without interfering with sprinting ability. For statistical analysis, the best score recorded between the two attempts was considered.

**Figure 1:**
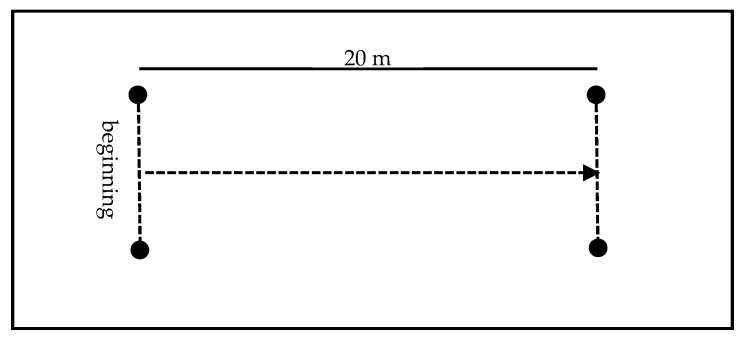
Linear Velocity—20 m. The arrow represents displacement.

The starting position was standardized: participants stood with their preferred foot forward and their upper limbs in a free position. The start was given by verbal command from the evaluator (“Ready—Go!”), to which the athlete had to react as quickly as possible, running the 20 m at maximum speed until crossing the finish line. The running time was timed from the moment of the initial movement until crossing the finish line (Figure 1).

It is assumed that a shorter execution time reflects better performance in the linear speed variable.

The choice of this protocol was based on a systematic review conducted by Altmann et al. [16], which concluded that the 20-m test has good levels of validity, reliability, and practical applicability in the context of football, being appropriate for athletes of different age groups and competitive levels.

#### 2.2.2. Direction Change Speed—5-0-5 Agility Test

The assessment of change-of-direction speed was performed using the 5-0-5 Agility Test, a protocol widely used in youth sports to measure the ability to decelerate, change direction, and accelerate in situations of high neuromuscular demand. This test has demonstrated good reliability and validity for youth football players [17].

The test was set up on synthetic turf using six markers, a tape measure, and a digital stopwatch. The course was outlined based on a starting line, an intermediate mark at 10 m, and a turning line at 15 m. The actual distance timed was 10 m (5 + 5 m), corresponding to the acceleration and return phase after the change of direction.

Each participant made two attempts with each dominant leg, i.e., they performed the test by reversing the direction of change on both sides (right foot and left foot), with a recovery interval of 1 to 2 min between repetitions. For the analysis, the best time obtained with each foot was considered.

**Figure 2:**
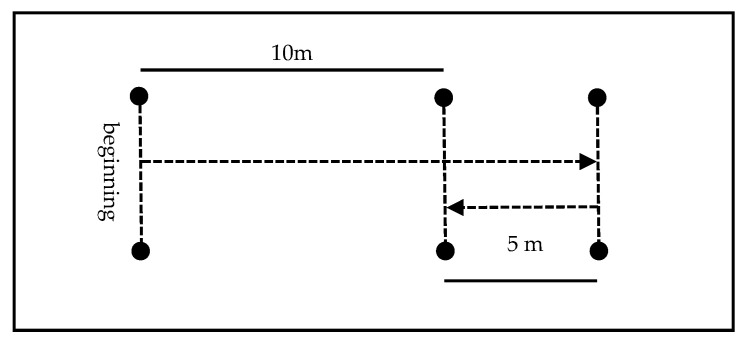
5-0-5 Agility Test. The arrow represents displacement.

The athletes started the test in a standing position, with their preferred foot in front of and behind the starting line. At the verbal signal from the evaluator (“Ready—Go!”), the athlete started running continuously, covering 15 m to the turning line, where one of the feet was required to clearly touch the marked line. Immediately after, the athlete made a 180° turn and sprinted back 5 m until passing the cones positioned at 10 m (timed zone) again (Figure 2).

The time recorded corresponds to the interval between the first pass through the 10 m and the second, after the change of direction (5 m + 5 m), with a shorter execution time reflecting better performance in the ability to change direction.

This protocol was selected based on evidence that the 5-0-5 Agility Test is sensitive to adaptations caused by speed, agility, and running technique training programs, being especially suitable for young populations and applicable in training and competition contexts [17].

### 2.3. Intervention

After an initial assessment session, the intervention group underwent a running technique training program lasting eight weeks, with the group completing one session per week, lasting 20–30 min each, held on Fridays at the beginning of training (Table 1).

For the planning of the sessions, we mainly used the “Grade I Coach Manual: Athletics”, and the book, *Conditioning Young Athletes* [18]. Training was organized into three phases: the warm-up phase, the running technique phase, and the playful exercises phase. The first phase of general warm-up, which sought to prepare the participants for the entire session by introducing exercises focused on warming up all segments, gradually begins to prepare the actions necessary for the session (Table 2).

The warm-up was carried out over 30 m (15 m + 15 m), performing the exercises twice, back and forth.

This was followed by a running technique phase, where specific running technique exercises were performed with the aim of improving running form and correcting aspects of posture, ground contact, lower and upper limb movement, and working on the motor coordination of the participants. As mentioned, the “Grade I Coach Manual” was the basis for the prescription, with the exercises and their progressions being based on it. The exercises are organized from the simplest to the most complex movements, starting with slower exercises with basic running components and progressing to more dynamic exercises closer to the movement of sprinting, since the last two running technique drills in the training plan already include sprint actions (20 m), they allow the practitioners to develop their running technique in a context of greater intensity and demand, bringing them closer to the reality of the game (Table 3).

Running technique exercises are performed over a total distance of 30 m (15 m + 15 m), with each exercise being performed twice. Those exercises that involve using the two lower limbs separately are performed once with each limb.

**Figure 3:**
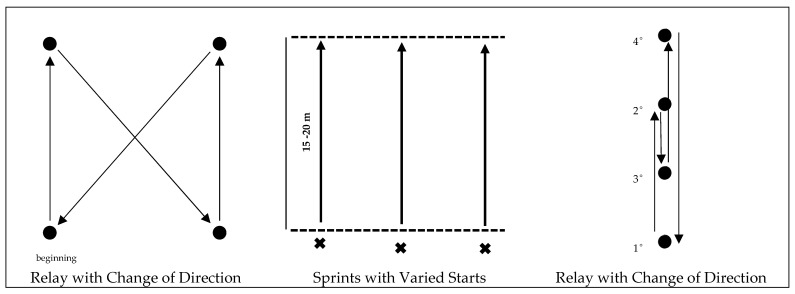
Playful exercises. Arrows represent movements, circles represent cones, and crosses mark players.

The sessions ended (phase III) with a more playful part, in which an exercise was carried out focusing on at least one of the motor skills considered fundamental for performance in the sport. Exercises were therefore planned with a focus on linear speed, speed with changes of direction, agility, and reaction time. The aim of this final phase of training was to integrate the running technique developed in the previous phases and bring it closer to the specific context of the sport, seeking to simulate common stimuli in football, but always emphasizing the efficiency of the running technique. Throughout the 8 weeks of intervention, a variety of exercises were carried out, most of which included running actions at speed, with or without changes of direction, starts in reaction to auditory or visual stimuli, and obstacle courses, among other exercises addressing the skills described above. All exercises were performed in a dynamic and competitive context, promoting intensity and the transfer of running technique to sport-specific situations, reinforcing the practical application of the content developed throughout each session (Figure 3).

The control group continued with their normal training routine during the 8 weeks.

To better understand each coach’s training method, the training plans of each team were observed during the 8 weeks of intervention, noting the type of exercises, intensity, and overall objectives of the sessions, to understand if there were moments that positively or negatively interfered with the development of the team’s speed and speed of change of direction.

The U15 team had 4 training sessions per week, each lasting 1 h and 30 min, and usually had 1 game on the weekend. The U17 team had 3 training sessions per week, each lasting 1 h and 30 min, and usually had 1 game on the weekend.

Both teams performed a 20-min strength circuit once per week, consisting of the same exercises at light to moderate intensity, as prescribed by the club’s performance department.

For the U15 team, which was in competitive season, normally when the game took place on Sunday, on Monday the training was carried out in a circuit with three stations, containing one station with recovery exercises, another with numerical superiority situations (e.g., 2v1; 3v2) and one for speed. The rest of the training consisted of specific football training, focusing on technical and tactical work with a focus on the weekend games, working on aspects such as coverage, transition, finishing, ball possession, and set pieces, among others.

For the U17 team, which was also in competitive season, they mostly performed specific football training, addressing technical and tactical components, working on aspects such as defensive transition, finishing, passing, set pieces, coverage, among others. This team also had the habit of doing speed work on Thursdays, given by the coach himself, working at speed, performing speed exercises with and without the ball.

### 2.4. Procedures

To implement this assessment, contact was made with a football club, to which the objective, methodology, and organization of the study were duly presented. After analysing the proposal, and by internal decision of the club, it was decided that the Under-15 teams would participate as the experimental group and the Under-17 teams would participate as the control group. All athletes involved and their families, as well as their coaches, were duly informed about the study procedures, and informed consent was obtained from all participants, in accordance with the ethical principles defined in the Declaration of Helsinki [19]. The performance assessment sessions were held at two different times: at the end of week 0 (pre-test) and at the end of week 9 (post-test). Between the assessment times, more precisely between weeks 1 and 8, a specific running technique program was implemented with the intervention group (Under-15), with no intervention being applied to the control group (Under-17), which maintained its regular training. All assessments took place on a synthetic turf field, ensuring uniformity in the conditions of performance. Test times were measured using manual timing, performed by previously trained evaluators to reduce measurement errors and ensure the reliability of the data collected.

### 2.5. Statistical Analysis

Descriptive statistics, including mean and standard deviation, median, and interquartile range, were calculated for the variables studied. The Shapiro–Wilk test (*n* < 50) and Levene’s test were used to verify the normality and homoscedasticity of the data, respectively. Given the small sample size [20] and the parametric assumptions were violated, including the central limit theorem’s applicability [21,22], non-parametric tests were selected. Group differences were assessed using the Mann–Whitney U test, both for within-group and between-group differences. To compare and identify possible differences in each group at different time points, the Wilcoxon signed-rank test was used. The effect size r (appropriate for the Wilcoxon test, which allows two paired groups to be compared) was calculated, and the following reference values were assumed: “small” effect ≥ 0.01, “medium” effect ≥ 0.3, and “large” effect ≥ 0.5 [20,23]. The significance level for rejecting the null hypothesis was set at 5%, and the analyses were performed using IBM SPSS Statistics V. 29.0.1.0.

## 3. Results

Table 4 presents descriptive statistics for the results of the 20-m and 5-0-5 Agility Test (right and left).

Looking at Table 4, we can see that, in the case of the intervention group there is a decrease in the average pre- and post-intervention in all tests; however, in the case of the control group, there was a regression in the results, with post-intervention values higher than pre-intervention values.

At baseline, there were differences between the groups in the 20-m test (*p* ≤ 0.001). After 8 weeks of intervention, no significant differences were found between the groups in the various tests performed (*p* ≥ 0.05) (Table 5). On the other hand, significant differences were found in the intervention group for the 5-0-5 Agility Test (right) (t = −2.590; *p* = 0.010; r = 0.669) and 5-0-5 Agility Test (left) (t = −1.948; *p* = 0.05; r = 0.503). The control group did not show significant differences in the 5-0-5 Agility Test for the right foot (*p* = 0.18) and left foot (*p* = 0.53) or in the 20-m test (*p* = 0.06), although the mean values seem to indicate that the results worsened.

## 4. Discussion

The main objective of this study was to analyse the effects of implementing an eight-week weekly running technique program on linear speed and change-of-direction speed in young football players, using the 20-m test and the 5-0-5 Agility Test.

The results of the 20-m test did not show statistically significant differences between the pre- and post-intervention moments in any of the groups analysed (intervention and control). However, a positive trend of improvement was observed in the intervention group (Under-15), with a reduction in the average execution time. On the other hand, in the control group (Under-17), although no statistically significant differences were recorded, a slight regression in performance was observed, with an increase in the average time.

It is important to note that, at the pre-intervention stage, the two groups showed significant differences in the 20-m test, showing an initial advantage for the control group. However, at the post-intervention stage, this difference was no longer statistically significant. This change can be interpreted as a result of improvements in the performance of the intervention group and, simultaneously, a decrease in the performance of the control group, which indirectly reinforces the effectiveness of the running technique program in maintaining or improving linear speed.

Although the data did not reach statistical significance in the 20 m test, the observed improvement trend in the intervention group may reflect neuromuscular adaptations due to the running technique program. The reduction in average time and the decrease in the difference between groups at the post-test suggest that even a low weekly frequency pro-gram (1x/week) can induce neuromuscular adaptations favourable to sprint performance, as indicated in the literature [24,25].

Regarding change-of-direction speed, the results of the 5-0-5 Agility Test revealed statistically significant differences in the intervention group, both in the right foot support and in the left foot support. In addition, the effect size was high for both supports, which reinforces the effectiveness of the intervention in improving this specific ability.

About intergroup differences, no statistically significant differences were observed, neither at the pre-intervention stage, nor at the post-intervention moment. The absence of differences between the groups, together with the significant improvements in the intervention group, can be explained by the effectiveness of the program and the possible physical decline in the control group.

A possible explanation for the regression in performance observed in the control group (U-17) may be related to the stage of the competitive season they were in at the time of the final assessment. The intervention group (U-15) still had four games to play, was in the middle of the competitive season, and was presumably in better shape. By contrast, the control group (U-17) was preparing for the last game of the season, which may have negatively influenced their physical performance levels due to the natural decline in physical abilities throughout the season, as demonstrated in several longitudinal studies with young athletes [13,26].

The results obtained are consistent with the previous research, such as that of Lupo et al. [11] and Martín-Moya et al. [12], who reported significant improvements in speed and agility after specific running technique interventions. The study by Lupo et al. [11], for example, applied a 12-week program with two weekly sessions, observing significant gains in both linear speed and ball-carrying speed. Martín-Moya et al. [12] implemented a 6-week plan with three weekly sessions of combined training (strength, running technique, and plyometrics), reporting consistent improvements in change of direction.

Unlike these studies, the present study used a reduced training frequency (only one session per week) and still achieved statistically significant improvements in agility, suggesting that the quality, specificity, and consistency of the stimulus may be as or more relevant than its frequency, especially when the goal is to improve motor patterns and the mechanical efficiency of running.

In addition to performance gains, running technique can also bring benefits in terms of injury prevention. The study by Silva et al. [8] showed that a six-week running technique program resulted in relevant biomechanical changes, such as reduced pelvic flexion at initial contact, decreased ipsilateral pelvic elevation and lateral flexion, and external thoracic rotation during sprinting—changes associated with a lower risk of hamstring injuries, which are particularly prevalent in young football players. These findings are supported by Hall et al. [27], who identified that non-contact injuries account for most occurrences in young football players, mainly affecting the lower limbs (75.3%).

In this context, the program developed in the present study may serve not only as a performance improvement tool but as an effective preventive strategy for reducing the risk of non-traumatic injuries.

### 4.1. Study Limitations

This study has some methodological limitations that should be considered when interpreting the results. First, the uneven age distribution between the groups (Under-15 vs. Under-17) may influence training responses and physiological adaptations. Due to logistical constraints imposed by the club, age-based group allocation was the only feasible option. To mitigate the influence of baseline differences, we focused on within-group (intra-group) analyses, which allow for a more accurate interpretation of changes over time. The small sample size (*n* = 15 per group) limits the statistical power and generalizability of the results. The limited weekly frequency of the intervention (1×/week) restricts the total training volume and possibly the maximum impact of the program. Finally, manual timers have a large percentage of human error. This applies most especially to how quickly or slowly the person taking the time reacts to starting and stopping the timer. This can introduce inconsistencies and ultimately make the recorded performance less precise. On the other hand, the use of electronic timing devices and systems, i.e., photoelectric cells or fully automated timing devices, can help to greatly improve the accuracy and reliability of the recorded times. These systems rely less on human involvement, therefore ensuring more consistent measurements and allowing for more valid comparisons between trials or participants. Also, they are most useful when precision is critical, such as in competitive sports or research where there could be an excessively large difference resulting because of minor timing differences. The absence of biomechanical or physiological measures (e.g., strength, power, body composition) limits our ability to explain the underlying mechanisms of the observed sprint improvements.

### 4.2. Practical Implications

Despite the limitations, the results indicate that the implementation of a structured and specific running technique program can be a practical and efficient tool for improving change-of-direction speed and, potentially, linear speed, even with limited resources and a reduced training frequency. This approach represents a viable alternative for coaches and clubs wishing to develop their athletes’ performance with low-cost and time-reduced interventions, without compromising the specificity of football training.

In addition, the benefits observed in injury prevention, based on the current literature, reinforce the importance of integrating multidisciplinary components (running technique, strength, mobility, etc.) into training programs for young athletes.

## 5. Conclusions

The results of this study show that the intervention group, which underwent an eight-week running technique training program, appears to show significant improvements in change-of-direction speed, as demonstrated by statistically significant differences in the 5-0-5 Agility Test (for both lower limbs). In addition, although no statistically significant differences were observed in the linear speed test (20 m), there appears to be a trend toward improvement in the performance of this group. By contrast, the control group not only failed to show improvements but seems to show a regression in results at the post-intervention stage, suggesting a possible decrease in physical condition or absence of specific stimuli throughout the period under analysis.

These findings support the effectiveness of the running technique program in improving change-of-direction speed in young football players, with possible benefits also in linear speed. In addition to the gains observed in physical performance, the current literature suggests that this type of intervention may also contribute to reducing the risk of injuries, especially in muscle structures with a high incidence of injuries in youth football contexts, such as the hamstrings. Thus, the systematic implementation of running technique sessions is not only an efficient strategy for optimizing sports performance, but is a potential preventive measure in the field of musculoskeletal health for athletes.

## Figures and Tables

**Table 1:** Running technique training program.

General Warm-up (30 m: 15 m out + 15 m back)	Run (2×)—Hopping—Arm Circles Forward—Arm Circles Backward—Lateral Shuffle—Crossover Lateral Shuffle (lead leg forward and backward)—Backward Running
Running Technique 2× each exercise (15 m + 15 m)	Ankle Skipping—Single-leg Skipping—Single-leg Skipping alternating every 2 step—Skipping alternating every 1 step—Low Skipping—Skipping alternating every 2 steps—Medium Skipping—Skipping alternating every 3 step—High Skipping—High Skipping + Acceleration (20 m)—Backward High Skipping + Acceleration (20 m)
Final exercise	Focus on components:—Speed—Speed with Change of Direction—Agility—Reaction Time

**Table 2:** Warm-up.

Exercise	Description
Jogging	Moderate intensity runs, going there and back twice.
Hopping	Alternate leg hops: while airborne, one lower limb is flexed at 90° while the other remains extended. The foot strike transitions progressively from heel to toe. Arms move in coordination with the legs.
Arm Circles	While moving with a bounding run, perform arm circles—both forward and backward.
Crossover Lateral Shuffle	Perform lateral movement, alternating the crossing of legs—once in front and once behind. When crossing in front, lift the knee.
Backward Running	Run backward, bringing heels up toward the glutes. Take long strides and apply force against the ground.

**Table 3:** Running Technique.

Exercise	Description
Tibiotarsal Skipping	Maintain the lower limbs extended, keeping the foot in dorsiflexion, promoting ground contact with the forefoot. The arms (upper limbs) move in coordination with the legs (lower limbs), flexed at approximately 90°.
Mono Skipping	One leg remains extended (as in tibiotarsal skipping), while the opposite leg performs a high skipping motion, lifting the knee to approximately waist height. Ground contact is made with the forefoot. Arm movement is coordinated with the legs.
Skipping (Low, Medium, and High)	Low—Fast, short ground contacts with the forefoot and low knee lift. The eyes are forward, trunk upright, pelvis high, and arm movement is short. Medium—Similar to the low skipping, but with moderate knee lift and medium arm movement. High—Forefoot contacts, fast and with the knee lifted to waist height. The posture remains upright, pelvis high, and arm movement is longer, like sprinting technique.
Skipping Switch (1 by 1; 2 by 2; 3 by 3)	1 by 1—starts with one of the Lower Limb on top with the knee bent, the athlete performs 3 bounces on the floor, changing the support after these 3 bounces, performs the sequence successively, Support on the ground should be quick and with the front third of the foot. The Lower Limb that touches the ground when the athlete is standing still should be extended, keeping the pelvis high and the Upper Limb coordinated with the Lower Limb. 2 by 2—starts standing still, with one of the Lower Limb on top with the knee bent, to the coach’s palm, performs 2 quick supports, returning to the starting position. The ground support should be quick and with the front third of the foot, the Lower Limb that touches the ground when the athlete is standing should be extended, keeping the pelvis high and the Upper Limb coordinated with the Lower Limb. Perform about 8–10 reps and switch the Lower Limb that is on top. 3 by 3—Start standing, with one of the Lower Limb on top with the knee bent, on the coach’s palm, perform 3 quick supports on the ground, when finished you are back in the starting position but with the other Lower Limb on top. The support on the ground should be quick and with the front third of the foot. The Lower Limb that touches the ground when the athlete is standing still should be extended, keeping the pelvis high and the Upper Limb coordinated with the Lower Limb. (when performed in motion, the exercise is the same, but the change of support is performed at the practitioner’s pace).
High Skipping with Acceleration	Begin with high skipping in place, transitioning immediately into a sprint upon verbal command.

**Table 4:** Descriptive statistics for speed and change-of-direction speed test results.

	Intervention Group		Control Group	
	Mean ± Standard Deviation			
	Pre-Intervention	Post-Intervention	Pre-Intervention	Post-Intervention
20 m	3.66 ± 0.12	3.61 ± 0.10	3.46 ± 0.16	3.54 ± 0.18
5-0-5 Agility Test (right)	2.45 ± 0.16	2.39 ± 0.12	2.44 ± 0.18	2.49 ± 0.15
5-0-5 Agility Test (left)	2.47 ± 0.18	2.39 ± 0.13	2.43 ± 0.14	2.47 ± 0.16

**Table 5:** Difference between groups and times for the results of the 20-m and 5-0-5 Agility Test.

	Intervention Group		Control Group		Pre			Post			
	Median (Interquartile Range)				*t* ^a^	*p* ^a^	Comparison of Pairs (Groups) ^a,c^	*t* ^a^	*p* ^a^	Comparison of Pairs (Groups) ^a,c^	Comparison of Pairs (Moments) ^b,c^
	Pre	Post	Pre	Post							
20 m	3.70 (0.11)	3.65 (0.13)	3.43 (0.15)	3.54 (0.21)	−3.407	*p ≤* 0.001	G1 ≠ G2	−1.372	*p =* 0.170	d	d
5-0-5 Agility Test (right)	2.46 (0.20)	2.36 (0.16)	2.40 (0.10)	2.47 (0.21)	−0.831	*p =* 0.406	d	−1.749	*p =* 0.080	d	pre ≠ post (Intervention group)
5-0-5 Agility Test (left)	2.45 (0.28)	2.37 (0.18)	2.40 (0.19)	2.41 (0.15)	−0.875	*p =* 0.381	d	−1.456	*p =* 0.145	d	pre ≠ post (Intervention group)

Note: ^a^, Kruskal–Wallis; ^b^, Wilcoxon; ^c^, Bonferroni correction; d, no differences detected; t, test value.

## Data Availability

The data that support the findings of this study are available from the corresponding author upon reasonable request. The data are not publicly available due to privacy and ethical restrictions.
